# AHANet: Adaptive Hybrid Attention Network for Alzheimer’s Disease Classification Using Brain Magnetic Resonance Imaging †

**DOI:** 10.3390/bioengineering10060714

**Published:** 2023-06-12

**Authors:** T. Illakiya, Karthik Ramamurthy, M. V. Siddharth, Rashmi Mishra, Ashish Udainiya

**Affiliations:** 1 School of Computer Science and Engineering, Vellore Institute of Technology, Chennai 600127, India; illakiya.t2020@vitstudent.ac.in; 2 Centre for Cyber Physical Systems, Vellore Institute of Technology, Chennai 600127, India; 3 School of Mechanical Engineering, Vellore Institute of Technology, Chennai 600127, India; siddharth.mv2019@vitstudent.ac.in; 4 School of Electronics Engineering, Vellore Institute of Technology, Chennai 600127, India; rashmi.mishra2019@vitstudent.ac.in (R.M.); ashish.udainiya2020@vitstudent.ac.in (A.U.)

**Keywords:** Alzheimer’s disease, convolutional neural network, deep learning, classification, magnetic resonance imaging

## Abstract

Alzheimer’s disease (AD) is a progressive neurological problem that causes brain atrophy and affects the memory and thinking skills of an individual. Accurate detection of AD has been a challenging research topic for a long time in the area of medical image processing. Detecting AD at its earliest stage is crucial for the successful treatment of the disease. The proposed Adaptive Hybrid Attention Network (AHANet) has two attention modules, namely Enhanced Non-Local Attention (ENLA) and Coordinate Attention. These modules extract global-level features and local-level features separately from the brain Magnetic Resonance Imaging (MRI), thereby boosting the feature extraction power of the network. The ENLA module extracts spatial and contextual information on a global scale while also capturing important long-range dependencies. The Coordinate Attention module captures local features from the input images. It embeds positional information into the channel attention mechanism for enhanced feature extraction. Moreover, an Adaptive Feature Aggregation (AFA) module is proposed to fuse features from the global and local levels in an effective way. As a result of incorporating the above architectural enhancements into the DenseNet architecture, the proposed network exhibited better performance compared to the existing works. The proposed network was trained and tested on the ADNI dataset, yielding a classification accuracy of 98.53%.

## 1. Introduction

AD is a neurodegenerative disease that causes the brain to shrink and damages neurons over time. It is the most common form of dementia and leads to a progressive decrease in thinking, abnormal behavior and social skills that affect a person’s capacity to live independently [1]. AD affects more than one in every nine adults aged 65 and above. It affects nearly 5.3% of those aged 65 to 74, 13.8% of those aged 75 to 84, and 34.6% of those aged 85 and above [2]. AD is caused by a mixture of genetic, behavioral and environmental factors that affect the brain over time. People with AD can be in the early, medium, or late phases of the disease. As the symptoms become more severe, it is difficult to slow down or stop their progression. If the condition is diagnosed in the preclinical stage known as Mild Cognitive Impairment (MCI), it is possible to slow or stop its rapid progression.

According to research studies, approximately 10–15% of MCI patients progress into the AD stage each year. Around 1–2% of healthy people developed AD during the same time period. This indicates early detection is a factor in yielding better outcomes [3]. The estimated occurrences of reversal from the MCI stage to the normal stage ranged from 4 to 15% in clinic-based research and from 29 to 55% in population-based studies. This confirms that MCI is a viable interventional stage for reversing or stopping the disease’s degenerative progression. Medications are given to treat the cognitive and non-cognitive symptoms of AD. A global effort is underway to identify new ways to diagnose and treat AD.

Memory, language and thinking difficulties are the common symptoms of AD. At the most basic level, the activity of neurons becomes interrupted [4]. The damage normally starts in the memory-controlling region of the brain. Furthermore, the loss of neurons extends to other parts of the brain. It finally causes the brain to shrink considerably during the late stages of the disease. Inability to communicate and make decisions, increased sensitivity to infections and fall injuries are a few of the long-term impacts of AD.

For the diagnosis of AD, neuroimaging technology is used as an important diagnostic tool. The various neuroimaging modalities are Computed Tomography (CT), structural Magnetic Resonance Imaging (sMRI), Positron Emission Tomography (PET), functional MRI (fMRI), and single-photon emission CT. sMRI and fMRI have become increasingly important in the diagnosis of structural and functional changes, respectively [5]. MRI is the gold-standard neuroimaging technique for assessing anatomical and functional changes from a pathological perspective. Due to its unique features, MRI has become the key imaging modality for diagnosing AD and detecting MCI in recent years. In the context of clinical screening for Alzheimer’s disease, the traditional methods using MRI have shown relatively satisfactory results for differentiating between AD and healthy controls (HC). MRI findings for MCI and AD appear quite similar, making it difficult to distinguish MCI from AD. Hence, this research work proposes an effective deep learning network based on DenseNet-169 architecture integrated with two attention modules, namely ENLA and Coordinate Attention. An AFA module is used to fuse the features in an effective way for precise classification.

## 2. Related Works

In this section, the different methods applied for the classification of AD based on medical imaging are reviewed in two categories: The first category analyzes machine learning approaches. The second category deals with the deep learning-based methods employed in the classification of AD.

### 2.1. Machine Learning-Based Methods

Traditional machine learning methods have various advantages with respect to the classification of AD. They require large amounts of data to understand the trends associated with Alzheimer’s detection. These insights have been utilized by researchers to address the issues associated with AD classification. The various machine learning algorithms used for AD classification include K-Nearest Neighbors (KNN), Decision Trees, Support Vector Machines (SVM), etc.

Machine learning uses handcrafted features for AD classification. For instance, Gao et al. developed a novel method that utilizes a gray-level co-occurrence matrix for feature extraction and an Extreme Learning Machine (ELM) for the classification of AD [6]. A similar approach was proposed by Sudharsan et al. that uses Informative Vector Machine (IVM), Regularized Extreme Learning Machine (RELM), and SVM for the classification of AD [7]. Additionally, Principal Component Analysis (PCA) was employed for feature selection and dimensionality reduction. Further, Yi et al. presented a method where morphometric and texture features were extracted and classification was performed by SVM with a Radial Basis Field (RBF) kernel [8].

A multilayer ensemble decision tree was presented by Naganjaneyulu et al. for the classification of AD [9]. A weighted feed-forward neural network along with an improved decision tree for feature selection were designed for the classification of AD. Another similar ensemble approach for early AD diagnosis was proposed by Rohini et al. Naive Bayes, KNN and SVM classifiers were combined for multiclass AD classification [10]. The work proposed by Cabrera-León et al. used resampling techniques to solve class imbalance and compared the non-neural ensemble networks with the counter propagation network for AD classification [11]. Another novel method based on a Gaussian discriminant analysis-based Computer-Aided Diagnosis (CAD) system was presented by Fang et al. [12]. Feature selection methods based on variance analysis and incremental analysis were employed for AD screening.

Undoubtedly, machine learning methods are utilized in most AD classification methods. However, researchers have shifted to deep learning algorithms for better performance in AD classification using neuroimaging data. The main reason is that deep learning methods provide better accuracy on diverse data. Furthermore, machine learning methods require domain knowledge for proper feature selection, whereas in deep learning methods, the important features are automatically extracted for precise classification.

### 2.2. Deep-Learning-Based Methods

Several research studies have utilized various deep learning approaches for the classification of AD. Convolutional Neural Networks (CNN) are widely utilized in image-based disease diagnosis because of the following: (1) They can handle a large number of contextual features, including pathological information. (2) The processing is hierarchical and makes use of spatial relationships across the input image. (3) They are also computationally efficient due to their use of special convolution, pooling, and parameter sharing operations.

The pre-trained CNN models are used in transfer learning, where the learned parameters from one model can be used as input parameters in another model to make predictions. It is most effective when the target data is similar to the input data. A substantial amount of work has been performed in the field of AD classification using transfer learning. Deep neural network architectures such as AlexNet, VGG16, ResNet, etc. have been successfully used for AD classification. For instance, Bae et al. incorporated a modified ResNet-50 architecture for binary AD classification [13]. Another similar approach presented by Yadav et al. employed the use of axial and sagittal slices of a brain scan in a custom 2D CNN architecture along with ResNet-50 for early AD classification [14]. Similarly, Sun et al. proposed a modified ResNet-50 architecture that employs spatial transformer networks (STN) and a non-local attention mechanism for early AD diagnosis [15]. Jain et al. proposed a method that uses the VGG-16 architecture for feature extraction. The final classification was performed using fully connected layers [16]. Similar to the previous methods, Jiang et al. proposed a method using VGG-16 for transfer learning. Lasso algorithm was utilized for feature selection, and it employed SVM for AD classification [17]. In an effort to improve accuracy, Kang et al. proposed a multi-modality approach that uses sMRI and Diffusion Tensor Imaging (DTI) images to detect AD using VGG-16 and an SVM classifier [18]. In addition to the previous methods, Shanmugam et al. presented a comparison between GoogleNet, AlexNet and ResNet-18 for the classification of AD and MCI. ResNet-18 was observed to perform better than the other architectures [19]. Another work based on the comparison of different pre-trained networks such as EfficientNetB0, DenseNet, ResNet-50, Xception, etc. for AD classification was proposed by Savaş [20]. From the results, it was inferred that EfficientNetB0 performed slightly better than the other architectures. In a similar effort, Ashraf et al. examined second-generation neural networks and spiking neural networks for AD classification [21]. It was inferred that DenseNet gave better results in terms of accuracy for the three-way classification of AD.

Convolutional neural networks are used for creating custom models because they are flexible and produce better results than pre-trained models. AbdulAzeem et al. developed a new five-layered customized CNN for the classification of AD. This work used data augmentation and adaptive thresholding for processing the images [22]. Similarly, Spasov et al. developed a feature extractor sub-network based on grouped and separable convolutions to perform AD classification [23]. Katabathula et al. proposed a dense CNN architecture that combines global shape representations with hippocampal segmentations for AD classification [24]. Moreover, the combined score of demographic information, genetic status, and standard cognitive tests was used along with MRI images. Basaia et al. proposed another method that uses data augmentation and standard convolutional layers in a custom 3D CNN instead of max pooling layers [25]. In contrast to the previous method, Li et al. presented an approach using residual blocks in the CNN for feature extraction to differentiate between the AD classes [26]. Basheera et al. designed a custom CNN architecture with five convolution layers for GM atrophy-based AD classification [27]. Later, they developed a novel CNN model to perform binary and multi-class classifications of AD [28]. They have incorporated inception and residual blocks in the CNN model for deeper feature extraction for early AD classification. The gray matter segmentation from slices was performed using Enhanced Independent Component Analysis (ECIA) [29]. Raju et al. designed a custom 3D CNN architecture to extract image features and adopted an SVM with an RBF kernel to perform AD classification [30]. Another work using 3D CNN along with SVM was presented by Feng et al. for the classification of AD [31]. Similarly, Shen et al. employed a method that used a custom CNN model to extract salient features. SVM was further employed to predict the chances of patients’ conversion from MCI to AD [32].

As single-modality data can only characterize a few of the degenerative alterations linked to AD, the performance of the classifier may be limited. Hence, considerable research work has been performed to implement classification techniques integrating multi-modal information. For instance, Huang et al. designed a VGG-like network to perform multimodal AD classification [33]. Another approach was proposed by Venugopalan et al., where stacked denoising auto-encoders were utilized to extract information from genetic and clinical data [34]. Furthermore, the authors used KNN, decision trees, random forests and SVMs as classifiers. Similarly, Zhou et al. developed a novel approach that used GAN and a Fully Convolutional Network (FCN) on 1.5 T and 3 T MRI scans [35]. To improve diagnostic performance, Yu et al. presented a novel Generative Adversarial Network (GAN) that uses 3D transposed convolution to generate MRI images [36]. In contrast to the previous methods, Han et al. designed two approaches for three-way AD classification [37]. In the first technique, the convolution module and the Cascade of Enhancement Nodes Broad Learning System (CEBLS) modules were combined for the classification of AD. In the subsequent method, the convolution module collected features while the Broad Learning System (BLS) module performed AD classification. To improve the results, Choi et al. presented a novel approach to utilizing a deep Ensemble Generalization Loss (EGL) for weight optimization to perform AD classification using an ensemble of many deep CNNs [38]. In addition, Zeng et al. employed a novel Deep Belief Network (DBN) that uses dropout and zero masking strategies to enhance the stability and generalization ability of the model [39]. Rashid et al. developed a novel architecture called the Biceph-Net module that is used in addition to 2D-CNN [40]. This module was employed in extracting the intra-slice and inter-slice information of the 2D MRI.

The wide use of convolutional neural networks to detect AD based on neuroimaging data is continuously improving the classification performance and has scope for further improvement.

### 2.3. Research Gaps

The proposed work addresses the following research gaps in the three-way classification of AD vs. MCI vs. HC.

1. Due to large semantic variations, it is challenging for a model to capture significant and distinct features of MCI and AD [41]. Local features were used in the majority of existing deep architectures that perform AD classification. Extraction of features on a global scale can support effective feature learning. Although few works have used multi-scale features, they lack the ability to efficiently exploit the information captured at different scales for classification.
2. In existing works, the local information from adjacent layers was directly combined for further processing. This may introduce some irrelevant background noise during the training process. Moreover, it makes the model difficult to distinguish subtle differences in the input images for effective feature extraction.
3. There is a need to identify the important features to classify MCI data as AD or HC. It has proven to be very challenging due to the complex and subtle differences in the MRI. Hence, it is necessary to utilize prominent and distinct multi-scale features extracted from the brain regions to attain accurate results.

### 2.4. Research Contributions

The following are the main contributions of the proposed work in the three-way classification of AD vs. MCI vs. HC.

4. The proposed work utilizes two attention blocks to extract prominent global and local features separately. The correlations between the multi-scale feature maps are extracted to strengthen the feature extraction process and ensure that important information is utilized effectively.
5. The Enhanced Non-Local Attention (ENLA) and Coordinate Attention modules identify and extract prominent global and local information from the brain regions. The ENLA block also consists of a residual link that is responsible for capturing channel-wise attention features. The Coordinate Attention module captures long-range dependencies and also retains spatial and positional information for improved performance.
6. We have also proposed an Adaptive Feature Aggregation (AFA) module that fuses the global and local features extracted at the prior level in an effective way. The global features guide the local features to focus on retaining spatial information for precise localization and improved learning capability. Moreover, it suppresses unnecessary background noise and utilizes only the important information for accurate classification.

## 3. Proposed Work

**Figure 1:**
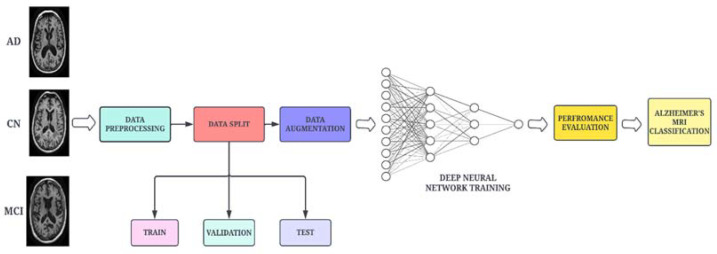
Schematic workflow of the proposed methodology.

A high-level architectural diagram of the proposed framework is illustrated in Figure 1. The proposed work uses the DenseNet-169 architecture, which is integrated with two attention modules and an adaptive feature aggregation module to fuse the features in an effective way. The augmented images are passed through the DenseNet-169 architecture to extract salient features. The output feature map is then propagated to the ENLA module and the Coordinate Attention module in parallel. The non-local attention module captures long-range dependencies (global features) through non-local operations, while the Coordinate Attention module captures cross-channel, direction-aware and position-sensitive information (local features). Moreover, the global and local features are passed into the adaptive feature aggregation module to harness multi-scale information from the network in a guided and efficient way. Finally, the categorical cross-entropy loss function is employed to measure the performance of the proposed network.

### 3.1. Adaptive Hybrid Attention Network (AHANet)

**Figure 2:**
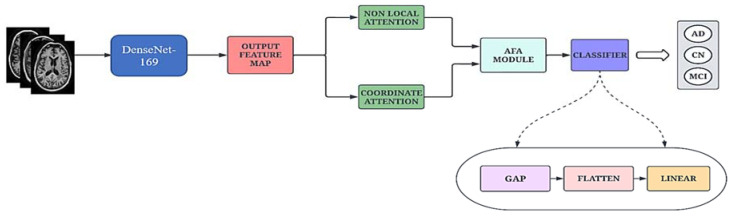
Schematic diagram of the proposed Adaptive Hybrid Attention Network.

The proposed network, AHANet, operates on the stack of T1 and T2 MRI images. The DenseNet-169 architecture was used due to its powerful feature extraction and feature propagation capabilities [42]. It is imperative to extract global and local features to strengthen the architecture and make it more robust. Therefore, we have employed ENLA and the Coordinate Attention module to effectively extract the salient global and local features, respectively. Furthermore, the AFA module adaptively fuses the features from the adjacent layers based on a squeeze-and-excitation operation to model prominent correlations. The schematic diagram of the proposed network is illustrated in Figure 2.

Finally, the output feature map from the AFA module is passed through a classifier block consisting of Global Average Pooling (GAP), Flatten and Linear layers for classification.

#### 3.1.1. Enhanced Non-Local Attention Module (ENLA)

**Figure 3:**
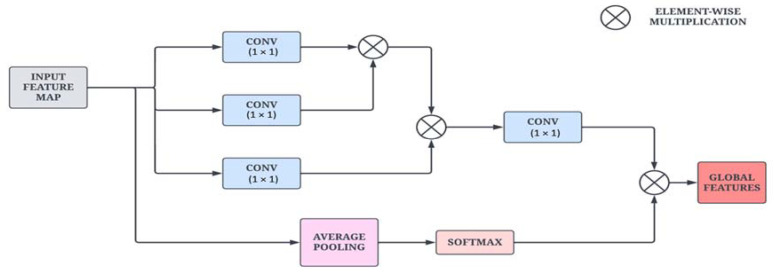
Architectural diagram of the Enhanced Non-Local Attention module.

To obtain global information from the input images, a non-local attention module is introduced. The architectural sketch of the proposed module is presented in Figure 3. The ENLA module captures long-range dependencies with the help of non-local operations. Furthermore, contextual information is also collected to enhance the pixel-wise representation power of the model. Given an input feature map x, the non-local operation is defined in Equation (1).

(1) $$y_{i}=\frac{1}{H\left(x\right)}\underset{\forall_{j}}{\sum }f\left(x_{i},\;x_{j}\right)g(x_{j})$$

where *x* and *y* are the input and output of the non-local attention block, *i* is the index of the output position, and *j* is the index of all the positions to be calculated. H is the normalization factor, which is defined in Equation (2).

(2) $$H\left(x\right)=\underset{\forall_{j}}{\sum }f\left(x_{i},\;x_{j}\right)$$

Given an input feature map $x_{i}$, the output feature map $y_{i}$ can be calculated using a softmax function along the dimension *j*. The correlation function $f\left(x_{i},\;x_{j}\right)$ is used to measure the similarity that is defined in Equation (3). The function $g(x_{j})$ computes a representation of the input signal at position *j*.

(3) $$f\left(x_{i},\;x_{j}\right)=\theta {\left(x_{i}\right)}^{Τ}\delta \left(x_{j}\right)$$

where $\theta \left(\cdot \right)$ and $\delta \left(\cdot \right)$ are feature transformations. Here, $\theta \left(x_{i}\right)=W_{\theta }x_{j}$ and $\delta \left(x_{j}\right)=W_{\delta }x_{j}$ are linear embeddings that are used to compute the representation of the input. In practice, a 1 × 1 convolutional layer is used to compute *θ* through matrix multiplication.

Additionally, a residual link with average pooling and softmax layers is added to compute channel-wise attention and strengthen feature propagation across the module. This mechanism weighs the channel information and adaptively recalibrates it to extract salient features. Due to the large number of filters from the previous layers in the DenseNet architecture, the channel attention mechanism acts as a normalization layer for the channel information. Moreover, the average pooling layer generates attention vectors, followed by the softmax layer that computes the attention coefficients.

#### 3.1.2. Coordinate Attention Module

**Figure 4:**
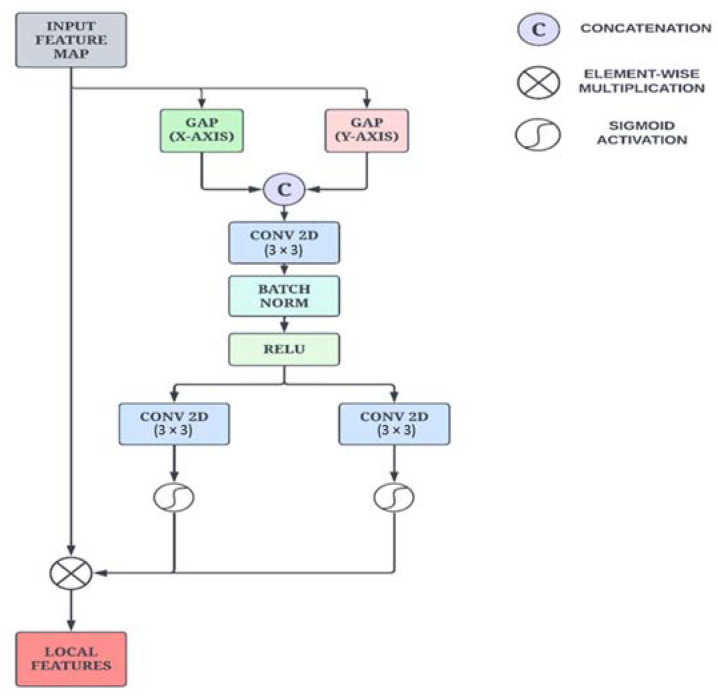
Schematic sketch of the Coordinate Attention module.

While the Enhanced Non-Local Attention block learns prominent global features from the input images, the Coordinate Attention module focuses on capturing precise local features along with long-range dependencies. Additionally, we have employed this particular module to preserve positional information, which is important for capturing spatial features. The mean values in each channel of the feature map are first calculated along the *x*-axis and *y*-axis using global average pooling. As illustrated in Figure 4, this is performed by using two spatial extents of pooling kernels (H, 1) and (1, W) to encode each channel along the horizontal and vertical coordinates, respectively. This is formulated in Equations (4) and (5).

(4) $$z_{c}^{h}\left(h\right)=\frac{1}{W}\underset{0\leq i\leq W}{\sum }x_{c}\left(h,\;i\right)$$

(5) $$z_{c}^{w}\left(w\right)=\frac{1}{H}\underset{0\leq j\leq H}{\sum }x_{c}\left(j,\;w\right)$$

The information from the vertical and horizontal directions is aggregated as per the two equations presented above, resulting in direction-aware feature maps. These transformations enable the attention module to capture long-range dependencies and retain positional information along spatial directions, thus improving the ability to locate features precisely.

The aggregated features are concatenated and propagated through a pointwise convolutional layer, which reduces the number of channels and is given by Equation (6).

(6) $$f=\delta \left(F_{1}\left(\left[z^{h},z^{w}\right]\right)\right)$$

where [·, ·] signifies concatenation along the spatial dimension, δ is a non-linear activation function, and $f\in \;{\mathbb{R}}^{C/r\times h}$ is the median feature map that encodes important spatial information along both the X direction and Y direction. Then the output feature map is split into the initial two groups along the spatial dimension.

Next, a convolution operation is performed in each group to transform the tensors with the same number of channels. Finally, after the sigmoid operation is applied, the raw feature maps are reweighed in the x and y directions, yielding Equations (7) and (8).

(7) $$g^{h}=\sigma \left(F_{h}\left(f^{h}\right)\right)$$

(8) $$g^{W}=\sigma (F_{W}(f^{W}))$$

where σ is the sigmoid function, and $f^{h}\;\mathrm{and}\;f^{W}$ are the output feature maps of each group before the transformation. The final feature map *y* is defined in Equation (9).

(9) $$y_{c}\left(i,\;j\right)=x_{c}\left(i,j\right)\times g_{c}^{h}\left(i\right)\times g_{c}^{W}\left(j\right)$$

where $g^{h}\;\mathrm{and}\;g^{W}$ are the output feature maps that are expanded and used as attention weights.

#### 3.1.3. Adaptive Feature Aggregation Module

The global-level and local-level features from the non-local attention module and the Coordinate Attention module are adaptively fused together to take advantage of complementary features. Global features contain information about shape descriptors and texture features, which can aid the local features in identifying important locations. As global features lack important spatial information and local features contain abundant spatial information, both are complementary to each other. As a result, we propose an adaptive feature aggregation module based on the squeeze-and-excitation (SE) layer to direct the feature fusion of adjacent layers.

**Figure 5:**
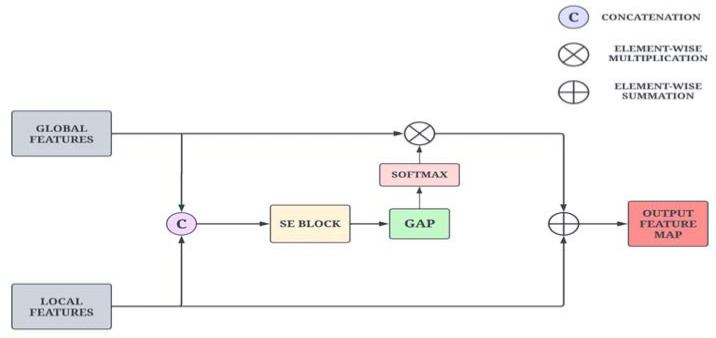
Schematic diagram of the Adaptive Feature Aggregation module.

As illustrated in Figure 5, the feature maps from the adjacent layers are first concatenated and passed through the SE layer to capture strong correlations across the channels. These feature maps are then fed into a pointwise convolution to reduce the number of filters. Then, the global average pooling layer is applied to extract channel-wise attention features on a global scale. The feature map is then propagated to the softmax function, which suppresses the irrelevant background noise and retains only the important information. Furthermore, the reweighted low-level features are added to the high-level features for improved feature representation power and precise localization. This operation is formulated in Equation (10).

(10) $$\eta^{\left(t\right)}=\eta_{h}^{\left(t+1\right)}⊕\;\left(\eta_{l}^{\left(t\right)}⊗\;\sigma \left(GAP\left(F\left(\eta_{f}^{\left(t\right)}\right)\right)\right)\right)$$

where $\eta_{f}^{\left(t\right)}=SE\left(\left[\eta_{l}^{\left(t\right)},\;\eta_{f}^{\left(t+1\right)}\right]\right)$, ⊕  and ⊗ represent element-wise summation and element-wise multiplication and F  denotes the 1 × 1 convolution layer.

## 4. Results

The effectiveness of the proposed network is evaluated in this section via ablation experiments. A comprehensive view of the dataset, experimentation and model training is presented.

### 4.1. Dataset Description

The data is taken from the ADNI database, which is made public on the website https://adni.loni.usc.edu/ (accessed on 7 April 2022). ADNI was launched in 2003 by the National Institute on Aging (NIA), the National Institute of Biomedical Imaging and Bioengineering (NIBIB), and the Food and Drugs Regulatory Agencies (FDA). The main goal of ADNI is to check the sequence of MRI, positron emission tomography (PET), other biomarkers, and clinical and neuropsychological assessments that may be combined to measure the progression of MCI and early AD. Subjects were recruited from more than 50 locations across the United States and Canada, providing written information agreed upon at the time of registration for image and DNA sampling, and completed questionnaires were approved by the Institutional Review Board (IRB) of each participating site. Table 1. shows the number of datasets and slices used for the implementation. A total of 930 subjects, including 220 AD, 456 MCI and 254 HC, were used in this work. In this research, we specifically focused on using T1-weighted images from the ADNI dataset.

### 4.2. Data Pre-Processing

**Figure 6:**
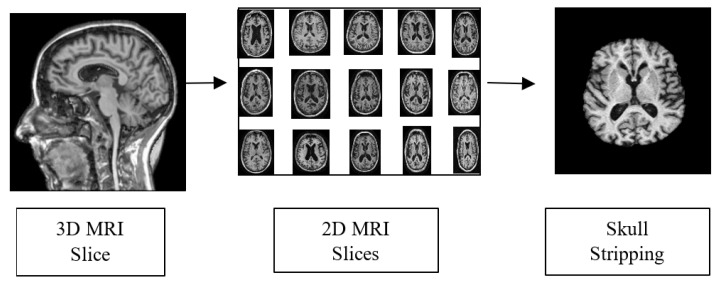
Pre-processing Pipeline.

To obtain accurate classification results, preprocessing steps are necessary to prepare the input data. The raw data in Neuroimaging Informatics Technology Initiative format is used as an input for the pre-processing phase. The 3D MRI voxels are converted into 2D slices for further processing. The pre-processing operations carried out in this work are presented in Figure 6. Skull striping is performed to remove the non-brain structure and unwanted portions from the scanned image. The scalp, skull and dura from the sMRI were removed using morphological structuring. Furthermore, the dataset was split into training, validation and testing sets in the ratio 60:20:20.

### 4.3. Data Augmentation

**Figure 7:**
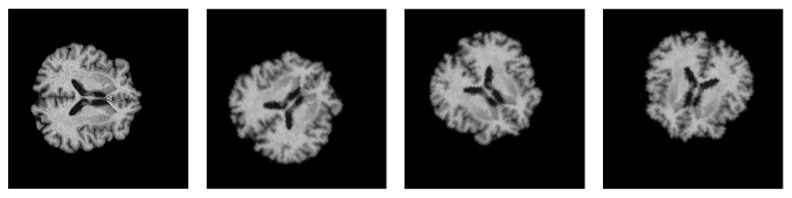
Visualization of the augmented brain MRI images.

To improve the generalizability of the proposed model and expose new variations during training, online augmentation was performed on the 2D samples. The dataset was augmented by applying random geometric transformations such as flipping and rotation. The parameter range for the random rotation function is in the range of 0° to 90°, and the probability of random horizontal and vertical flips is set to 50% each. Figure 7 depicts the augmented brain MRI images. Furthermore, class imbalance was also solved using data augmentation. All the transformations were carried out using the Torchvision library.

### 4.4. Environment Setup

All image pre-processing tasks and training of the proposed model were implemented on the AWS EC2 instance using the Pytorch framework with a 16 GB NVIDIA T4 GPU. The proposed work utilized system resources consisting of the Ubuntu 20.04 operating system, 4 AMD vCPUs, and 32 GB of RAM. The model employs Stochastic Gradient Descent (SGD) with a learning rate of 1 × 10^−3^, momentum of 0.9, and weight decay of 1 × 10^−3^ for optimal performance and fast convergence. The proposed network was trained and validated for 50 epochs. To decrease the training time of the network without compromising on performance, we employed mixed-precision training using the AMP CUDA library. Moreover, to prevent the problem of vanishing gradients, gradient scaling was employed during backpropagation.

### 4.5. Hyperparameter Tuning

The Grid Search algorithm on the Ray Tune framework was utilized to perform hyperparameter tuning. The experiment was setup with three hyperparameters in the model: (1) learning rate of the optimizer; (2) weight decay of the optimizer and (3) batch size. Optimal tuning was attained by iterating through the search space of parameter values in the specified range: the learning rate of the optimizer was set between 1 × 10^−1^ and 1 × 10^−5^; weight decay was one of the following: 0, 1 × 10^−3^, 1 × 10^−4^, 1 × 10^−5^; batch size was either 32 or 64. The proposed network resulted in optimal convergence with a learning rate of 1 × 10^−3^, a decay rate of 1 × 10^−3^, and a batch size of 32.

### 4.6. Ablation Studies

This section explores the effectiveness of the three important blocks for performance enhancement in the DenseNet architecture: (1) the Enhanced Non-Local Attention layer, (2) the Coordinate Attention layer and (3) the Adaptive Feature Aggregation layer.

#### 4.6.1. Analysis of the DenseNet-169 Network

**Figure 8:**
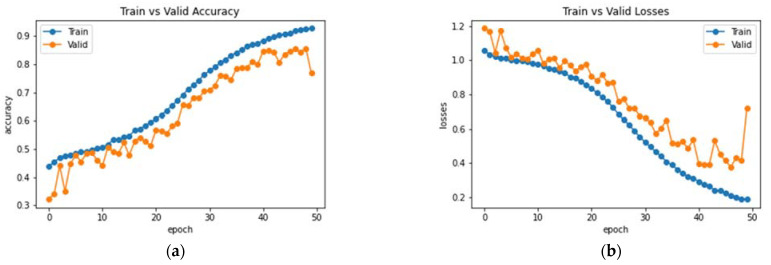
Analysis of DenseNet-169 (**a**) Accuracy (**b**) Loss.

**Figure 9:**
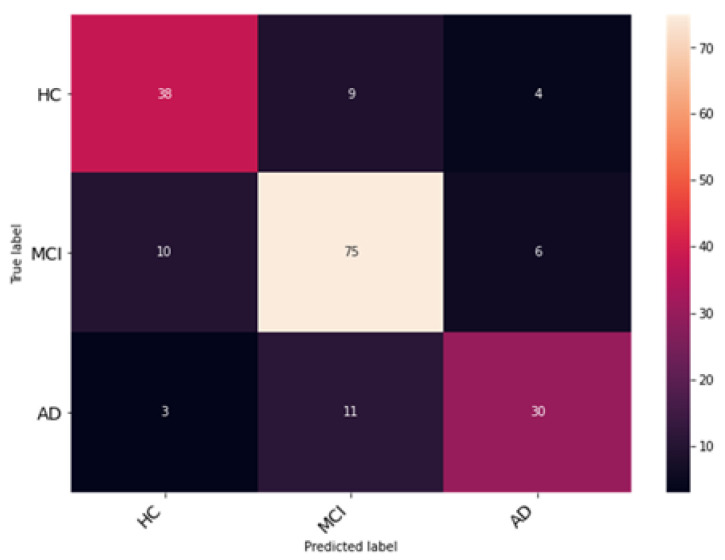
Confusion matrix on the test dataset.

This subsection analyzes the performance of the baseline DenseNet-169 architecture. Initially, the model was trained and validated for 50 epochs on the MRI dataset. The resultant observations of the model training processes are presented in Figure 8. An accuracy of 77% was obtained on the testing set with the baseline model. Furthermore, the average precision, F1 score and recall were 75.81%, 75.66% and 76.37%, respectively. Class-wise metrics are also presented in Table 2. To visualize the performance of the network on the dataset, a confusion matrix is also illustrated in Figure 9.

#### 4.6.2. Effectiveness of the Enhanced Non-Local Attention Module

**Figure 10:**
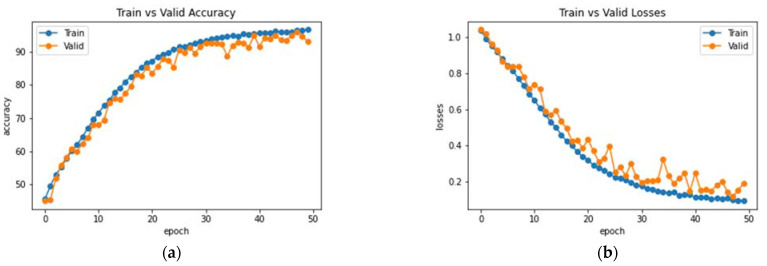
Analysis of DenseNet-169 with the Enhanced Non-Local Attention Module (**a**) Accuracy (**b**) Loss.

**Figure 11:**
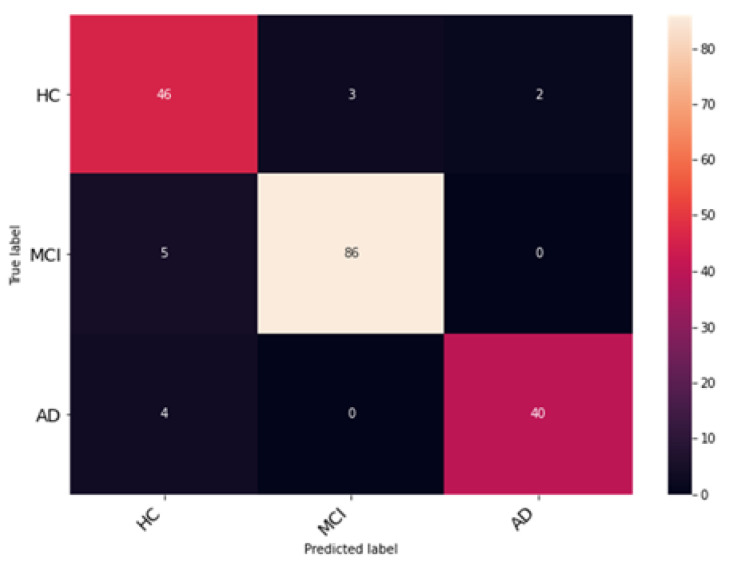
Examination of classification accuracy of the test dataset using the confusion matrix.

The Enhanced Non-Local Attention module is proposed in this work to extract salient global features and contextual information from the input images. Channel-wise attention is computed using the global average pooling and softmax layers, which are added using a residual link. The performance of DenseNet with the Enhanced Non-Local Attention block is analyzed, and the observations are presented in Figure 10. The model was trained and validated for 50 epochs. The obtained accuracy, precision, F1 score and recall were 93%, 92.11%, 92.78% and 93.27% on the test data. A confusion matrix is also shown in Figure 11 to visualize the performance of the network on the dataset. Further, class-wise metrics are also presented in Table 3.

#### 4.6.3. Effectiveness of the Coordinate Attention Module

**Figure 12:**
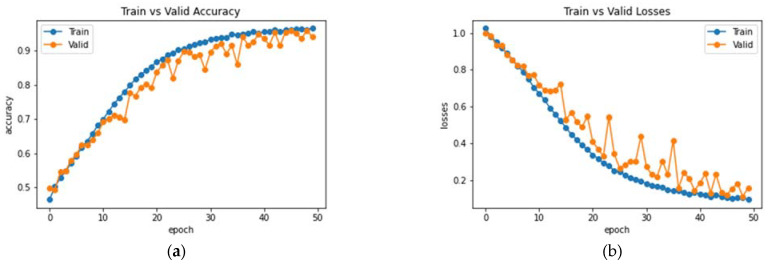
Analysis of DenseNet-169 with the Coordinate Attention Module (**a**) Accuracy (**b**) Loss.

**Figure 13:**
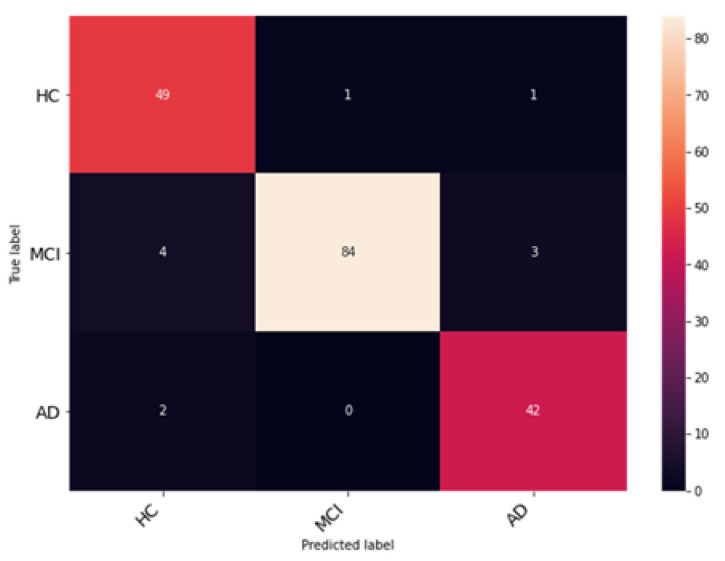
Analysis of classification accuracy through the confusion matrix on the test dataset.

In this experiment, the Coordinate Attention module is added to the DenseNet architecture to validate and analyze its performance. The Coordinate Attention module helps to improve the extraction and preservation of spatial information while also capturing long-range dependencies. The resultant observations of the model training are illustrated in Figure 12. The network was trained and validated for 50 epochs. An accuracy of 94% was obtained on the testing dataset. The average precision, F1 score and recall obtained were 94.33%, 93.67% and 93.98%. The class-wise metrics and confusion matrix are presented in Table 4 and Figure 13, respectively.

#### 4.6.4. Analysis of the Proposed Adaptive Hybrid Attention Network

The proposed network is a modified DenseNet-169 architecture with three additional modules: (1) an Enhanced Non-Local Attention module; (2) a Coordinate Attention module and (3) an Adaptive Feature Aggregation module. The attention modules were added to capture global and local features separately to improve the robustness and overall performance of the network. It is imperative to fuse the feature maps from the attention modules in an adaptive and effective way to take advantage of complementary features. The global features guide the local features to locate prominent features precisely. Furthermore, irrelevant background noise in the feature maps is suppressed with the help of the sigmoid function.

**Figure 14:**
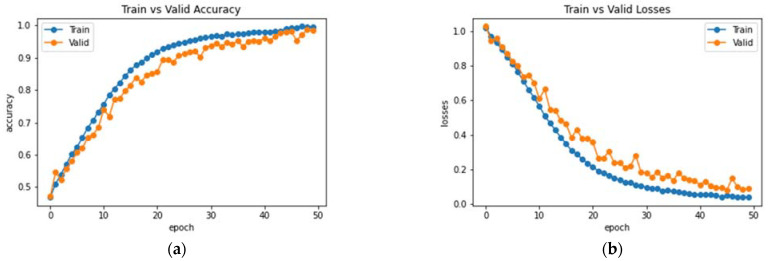
Analysis of the proposed AHANet (**a**) Accuracy (**b**) Loss.

**Figure 15:**
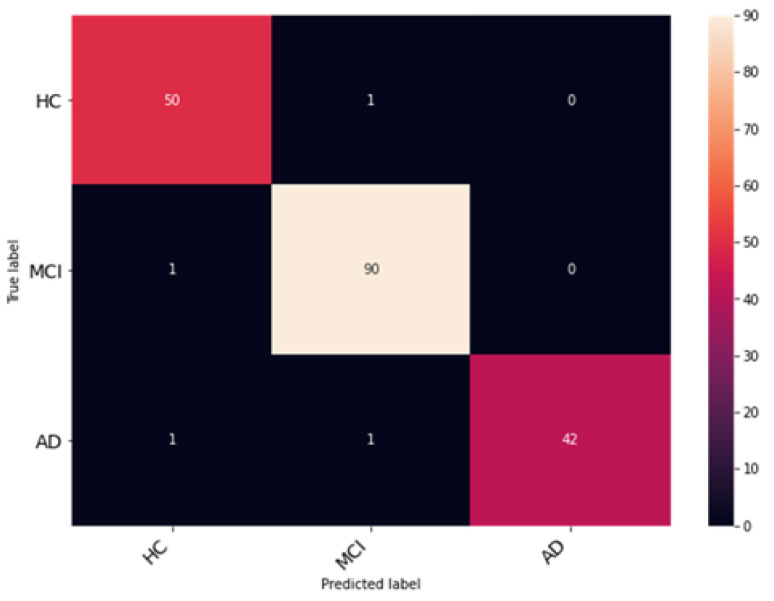
Evaluation of classification performance using confusion matrices for classes HC, MCI, and AD.

The proposed network was trained for 75 epochs, and it converged properly, as illustrated in Figure 14. The resultant accuracy obtained from the test data is 98.53%. The average precision, recall and F1 score obtained were 98.13%, 98.65%, 98.53%. Furthermore, class-wise metrics are tabulated, which can be seen in Table 5. To visualize the true positives, false positives, true negatives and false negatives, a confusion matrix is presented in Figure 15.

To quantify the usefulness of each enhancement, the blocks were gradually added to the DenseNet-169 architecture and trained. The results of this evaluation are tabulated in Table 6.

#### 4.6.5. Effectiveness of the Combined Attention Modules

Convolutional Block Attention Module (CBAM) and Squeeze and Excitation (SE) blocks are attention mechanisms used to enhance convolutional neural networks (CNNs) performance by focusing on informative features and suppressing less relevant ones. CBAM integrates both spatial and channel attention mechanisms into a single module. By combining these two attention mechanisms, CBAM effectively models both spatial and channel-wise dependencies in the feature maps. The SE block focuses on modeling channel-wise dependencies. It consists of two main steps: squeeze and excitation. In the squeeze step, global average pooling is applied to the input feature map to obtain a channel descriptor vector. In the excitation step, a fully connected network (usually with a few hidden layers) is used to model channel interdependencies. The output of the fully connected network is a set of channel-wise weights, which are applied to the input feature map to recalibrate channel-wise information. An experiment was performed to find the effectiveness of the CBAM and SE with DenseNet-169 as the base network in the classification of AD. The accuracy obtained by CBAM and SE is tabulated in Table 7.

Overall, the performance analysis demonstrates that the proposed work, with its combination of attention modules, outperforms CBAM and SE blocks with DenseNet-169 in terms of classification accuracy. The incorporation of multiple attention mechanisms effectively captures both global and local information, resulting in better classification performance.

## 5. Discussion

This section presents a comparison of the proposed work with state-of-the-art architecture and existing research. Moreover, all the models compared below were re-implemented for the ADNI dataset. The results were demonstrated on the test set common to all the experiments.

### 5.1. Comparison with the State-of-the-Art Networks

This section presents a performance comparison of the proposed CNN with state-of-the-art classification models on the ADNI dataset. The results of the models on the test set are tabulated below in Table 7. All the methods were implemented and run over the dataset with the same data distribution to generate these results. The pre-trained architectures were fine-tuned to adapt to this dataset. The proposed AHANet outperformed all the other models by a large margin.

**Figure 16:**
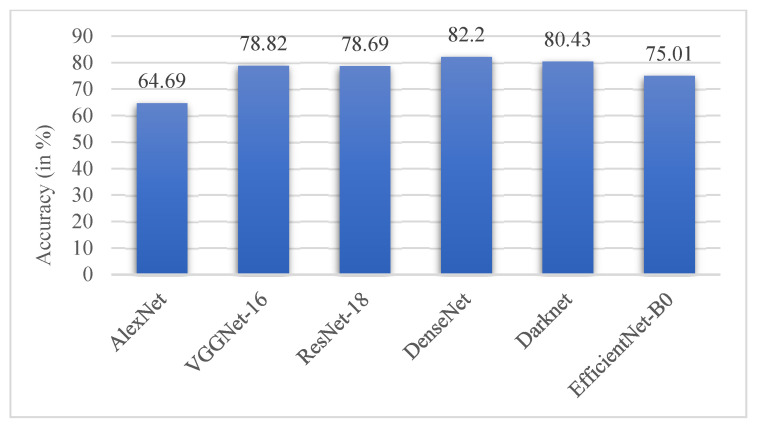
Comparison chart of the performance of state-of-the-art architectures.

Of all the architectures compared, DenseNet performed the best with an accuracy of 82.2%, followed by DarkNet with an accuracy of 80.43%. It could be inferred that AlexNet performed poorly on the ADNI dataset with a low accuracy score of 64.69%, as illustrated in Figure 16. The performance of the proposed AHANet managed to improve on DenseNet by 16.33%. On the whole, it could be inferred from Table 8 that AHANet outperformed the existing state-of-the-art architectures by a large margin.

### 5.2. Performance Analysis with the Existing Research Works

The performance of the proposed approach is compared against the existing work for Alzheimer’s detection using brain MRI images. We have included the works that have specifically employed the same ADNI dataset for a fair comparison. The compared works had classified the brain MRI into three classes (HC, MCI and AD).

It is to be noted that even though CNN architectures, such as DenseNet and EfficientNet, perform well for most vision tasks, they require additional customizations to capture and extract important and complex features. Table 9 presents a comparison of the proposed method with existing works. For a fair comparison between the proposed work and other AD classification studies, the related works have also performed multi-class (HC–MCI–AD) classification on the same ADNI dataset with AI techniques.

The classification accuracy reported by the transfer learning approaches is in the range of 92.9–95.7%. Although transfer learning is a powerful approach, especially to handle small datasets and prevent overfitting, modifications are required to make the model more robust. The classification accuracy reported by custom CNN approaches using 2D data and 3D data is in the range of 86.7–95.61% and 92.11–97.77%, respectively. In this proposed work, attention modules are added to strengthen feature extraction and the performance of the network. Furthermore, a novel feature aggregation block is proposed to fuse features from adjacent layers effectively. The proposed work outperforms all the compared works in terms of overall performance.

### 5.3. Limitations and Future Works

This section highlights the limitations of the proposed work and provides an overview of potential areas for future research and improvement.

- As compared to a single MRI modality, multimodal imaging data can provide more information, resulting in better classification results. Thus, future studies will address multimodal brain data, such as fMRI, PET, etc.
- In this study, all datasets were obtained from the ADNI. Nevertheless, we could benefit from additional data to account for even more feature variation. We plan to expand this study to include more data sources to increase the sample size.
- A potential direction for future work in this research article could be to explore the utilization of eXplainable Artificial Intelligence (XAI) techniques to elucidate the interpretation of the global and local features learned by the key components of AHANet, namely the ENLA layer, the Coordinate Attention layer, and the AFA layer. This would enhance our understanding of the model’s decision-making process and provide valuable insights into its contribution to AD classification.

## 6. Conclusions

This research presents a novel attention-based adaptive feature fusion framework for the classification of AD and MCI. Most of the existing work has overlooked the importance of extracting global and local features separately. Therefore, we have proposed AHANet to extract salient features and further adaptively fuse them to take advantage of the complementary attributes of the attention modules. The ENLA module captures features on a global scale, while the Coordinate Attention module focuses on capturing spatial features for precise localization. The above-mentioned modules enhance the feature representation power of the network and improve generalizability. Designing the novel AFA module to fuse features adaptively is a notable highlight of this work. The proposed AHANet outperforms the existing methods with an accuracy of 98.53%. It also achieved an average precision, recall and F1 score of 98.33%, 98.65% and 98.53%, respectively. As future work, this research offers rich scope to expand into other disease detection tasks using different imaging modalities. Additionally, the proposed modules can also be utilized for tasks such as semantic segmentation and object detection.

## Figures and Tables

**Table 1:** Details of the ADNI subjects utilized in the proposed work.

Diagnostic Type	Number of Subjects	Number of 2D Slices
AD	220	37,400
MCI	456	77,520
HC	254	43,180

**Table 2:** Resultant class-wise metrics for DenseNet-169.

Class	Precision in (%)	Recall in (%)	F1-Score in (%)
HC	75.0	75.0	75.0
MCI	82.0	79.0	81.0
AD	68.0	75.0	71.0

**Table 3:** Resultant class-wise metrics for DenseNet-169 with the ENLA module.

Class	Precision in (%)	Recall in (%)	F1-Score in (%)
HC	90.0	84.0	87.0
MCI	95.0	97.0	96.0
AD	91.0	95.0	93.0

**Table 4:** Resultant class-wise metrics for DenseNet-169 with the Coordinate Attention module.

Class	Precision in (%)	Recall in (%)	F1-Score in (%)
HC	96.0	89.0	92.0
MCI	92.0	99.0	95.0
AD	95.0	91.0	93.0

**Table 5:** Resultant class-wise metrics for the proposed AHANet.

Class	Precision in (%)	Recall in (%)	F1-Score in (%)
HC	98.0	98.0	98.0
MCI	99.0	98.0	99.0
AD	95.0	99.0	97.0

**Table 6:** Analysis of AHANet and DenseNet-169 with the proposed modifications.

Architecture	Model Parameters	Accuracy in (%)
DenseNet-169	18 M	82.2
DenseNet-169 with the ENLA Module	22 M	93.48
DenseNet-169 with the Coordinate Attention Module	22 M	94.38
DenseNet-169 with the ENLA and Coordinate Attention Module	32 M	94.96
AHANet (Proposed)	32 M	98.53

**Table 7:** Performance analysis of the CBAM, SE and proposed work.

Architecture	Accuracy in (%)
DenseNet-169 with CBAM	91.03
DenseNet-169 with SE	89.17
Proposed work	98.53

**Table 8:** Quantitative performance analysis of the proposed architecture with state-of-the-art methods for AD classification.

S. No	Model Trained	Number of Trainable Parameters	Accuracy in (%)
1	AlexNet	57 M	64.69
2	EfficientNet-B0	4 M	75.01
3	ResNet-18	11 M	78.69
4	VGGNet-16	134 M	78.82
5	DarkNet	26 M	80.43
6	DenseNet-169	12 M	82.2
7	AHANet (Proposed)	32 M	98.53

**Table 9:** Performance comparison of the proposed method with similar methods for detecting Alzheimer’s using MRI images.

Source	Method	Accuracy in (%)
Jain et al., 2018 [16]	VGG-16	95.7
Savas, 2021 [20]	EfficientNetB3	92.98
Feng et al., 2020 [31]	3D-CNN–SVM	92.11
Basheera et al., 2019 [27]	Custom 2D CNN	86.7
Basheera et al., 2020 [28]	Custom 2D CNN	86.7
Basheera et al., 2021 [29]	Custom 2D CNN	95.61
Raju et al., 2020 [30]	Custom 3D CNN	97.77
Choi et al., 2020 [38]	Ensemble of Deep CNNs	93.84
Venugopalan et al., 2021 [34]	Custom 3D CNN	88.0
Proposed Work	AHANet	98.53

## Data Availability

Data used in the preparation of this article were obtained from the Alzheimer’s Disease Neuroimaging Initiative (ADNI) database (adni.loni.usc.edu). As such, the investigators within ADNI contributed to the design and implementation of ADNI and/or provided data but did not participate in the analysis or writing of this report. A complete listing of ADNI investigators can be found at: http://adni.loni.usc.edu/wp-content/uploads/how_to_apply/ADNI_Acknowledgement_List.pdf. The ADNI dataset analyzed in this research work is available at https://adni.loni.usc.edu/ (accessed on 7 April 2022).
